# Scope of SARS-CoV-2 variants, mutations, and vaccine technologies

**DOI:** 10.1186/s43162-022-00121-z

**Published:** 2022-03-26

**Authors:** Josephine Wambani, Patrick Okoth

**Affiliations:** 1 Kenya Medical Research Institute (KEMRI) HIV Laboratory-Alupe, P.O Box 3-50400, Busia, Kenya; 2 Department of Medical Laboratory Sciences, School of Public Health, Biomedical Sciences and Technology, Masinde Muliro University of Science and Technology, P.O Box 190, Kakamega, 50100 Kenya; 3 Department of Biological Sciences, School of Natural Sciences, Masinde Muliro University of Science and Technology, P. O Box 190, Kakamega, 50100 Kenya

**Keywords:** Vaccination, SARS-CoV-2 variants, Mutations, Vaccine technologies

## Abstract

**Background:**

The COVID-19 pandemic is caused by the severe acute respiratory syndrome coronavirus 2 (SARS-CoV-2). SARS-CoV-2 is disseminated by respiratory aerosols. The virus uses the spike protein to target epithelial cells by binding to the ACE2 receptor on the host cells. As a result, effective vaccines must target the viral spike glycoprotein. However, the appearance of an Omicron variant with 32 mutations in its spike protein raises questions about the vaccine’s efficacy. Vaccines are critical in boosting immunity, lowering COVID-19-related illnesses, reducing the infectious burden on the healthcare system, and reducing economic loss, according to current data. An efficient vaccination campaign is projected to increase innate and adaptive immune responses, offering better protection against SARS-CoV-2 variants.

**Main body:**

The presence of altered SARS-CoV-2 variants circulating around the world puts the effectiveness of vaccines already on the market at risk. The problem is made even worse by the Omicron variant, which has 32 mutations in its spike protein. Experts are currently examining the potential consequences of commercial vaccines on variants. However, there are worries about the vaccines’ safety, the protection they provide, and whether future structural changes are required for these vaccines to be more effective. As a result of these concerns, new vaccines based on modern technology should be developed to guard against the growing SARS-CoV-2 variations.

**Conclusion:**

The choice of a particular vaccine is influenced by several factors including mode of action, storage conditions, group of the vaccinee, immune response mounted, cost, dosage protocol, age, and side effects. Currently, seven SARS-CoV-2 vaccine platforms have been developed. This comprises of inactivated viruses, messenger RNA (mRNA), DNA vaccines, protein subunits, nonreplicating and replicating vector viral-like particles (VLP), and live attenuated vaccines. This review focuses on the SARS-CoV-2 mutations, variants of concern (VOCs), and advances in vaccine technologies.

## Background

SARS-CoV-2 was first reported in Wuhan, China in December 2019 [1–3]. It is believed that the virus was transmitted to humans from an unknown animal reservoir [4, 5]. The virus has claimed a lot of lives globally and by December 2020 over 1.4 million people had succumbed to the disease and more than 6.35 million people had been infected with the virus [6]. SARS-CoV-2 has been linked to RaTG13, according to reports (a bat coronavirus). The SARS-CoV-2 spike protein is 97.4% similar to RaTG13. Its similarities to SARS-CoV and the Middle East respiratory syndrome coronavirus (MERS-CoV) are only 76% and 35%, respectively [3, 6–12].

The SARS-CoV-2 virus is a positive-strand RNA virus having genome size ranging between 27 and 31 kb [13–17]. These viruses are of the Coronaviridae family and currently 5 VOCs exist including the Omicron, Beta, Alpha, Gamma, and Delta [2, 10, 13]. Beta and Alpha VOCs are known to infect human beings [2, 10]. Coronaviruses infecting humans include human coronavirus NL63, human coronavirus 229E, coronavirus, human corona-virus HKU1, MERS-CoV, and SARS-CoV-2. Human coronavirus 229E was first identified and isolated in 1960s, SARS-CoV in 2002, human coronavirus NL63 in 2004, human corona-virus HKU1 in 2005, and MERS-CoV in 2012 [8, 10, 18]. Evolutionary analysis indicated that SARS-CoV-2 had close proximity to the SARS-CoV virus [2].

The virus spike glycoprotein binds to the ACE2 receptor on epithelial cells during infection [6, 19]. The spike glycoprotein interacts with cellular proteases after binding, causing it to be cleaved, allowing the virus to enter the cell [3, 6]. The viral genome is subsequently released into the cytoplasm, where it is translated by the host cell machinery, producing viral proteases, helicase enzyme, and RNA-dependent RNA polymerase (RdRp). RdRp is involved in viral genome replication and structural protein translation [6].

The rapid emergence of the SARS-CoV-2 virus necessitated the development of vaccines targeting the spike protein in an unprecedented timeframe [20]. Preclinical data from MERS-CoV and SARS-CoV research were used to develop novel vaccines [21]. Preparation processes from past vaccination research were used during development, and data from preclinical and toxicological studies were used in some instances [20]. This information was made public during the first phase of SARS-CoV-2 clinical trials, which took place in March 2021. Phase I and phase II investigations were undertaken at the same time in this preclinical research, followed by phase III studies. This came after a thorough examination of the positive phase I and phase II data [20]. Various vaccine production methods have been used to date, ranging from traditional to those that are being used in people for the first time [22]. Vaccines take about 10–15 years to develop and test before they are authorized for clinical use [23]. Different methodologies were employed in the manufacture of safe and highly effective vaccines for SARS-CoV-2 at a surprising pace, based on readily available data [24].

At the very least, the properties outlined in this article must be included in an ideal SARS-CoV-2 vaccine. It should be able to elicit long-lasting protective immune responses in everyone, regardless of underlying conditions, age, sex, breastfeeding status, immune state, and the lack of the ability to create pulmonary immunopathology or antibody-dependent enhancement [6]. It should also be thermostable and immune-stimulating [6]. SARS-CoV-2 mutations, variations of concern (VOCs), and developments in vaccination technology were the subject of this review study. This article also includes extensive background information on the SARS-CoV-2 vaccine’s development.

## Background information about SARS-CoV-2 vaccine developments

**Fig. 1:**
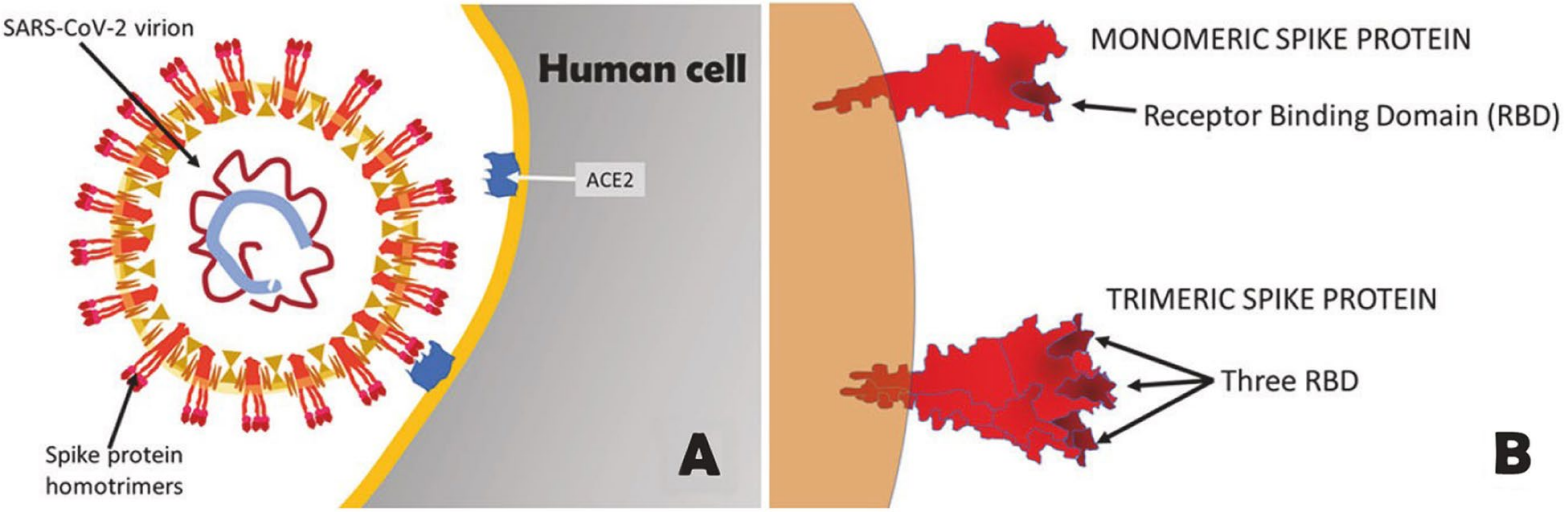
Schematic representation of SARS-CoV-2 and Spike glycoprotein [17]

The spike protein is a target for vaccine development because it mediates the virus’s contact with the host cells [1, 25]. The spike glycoprotein is important for virus attachment, entry, and subsequent neutralizing antibody production [14, 16]. This spike protein is located on the surface of the SARS-CoV-2 virus and has a role in virus attachment and entrance into host cells, making it a prime target for neutralizing antibodies [1, 9, 26]. As a result, when creating vaccines against the virus, the spike protein is chosen as the target antigen of choice, and it can be given in a variety of ways [9]. There are two types of approaches: gene-based and protein-based. The sort of spike design employed in this method is critical to its success since it can affect manufacturability, immunogenicity, vaccination effectiveness, biophysical, and antigenic characteristics [1, 27]. The spike protein has the surface ectodomain (S1) and the receptor-binding domain (RBD) in some designs, or the transmembrane domain (S2) or the ectodomain (S1) in others [1]. RBD is essential for viral attachment to host cells through ACE2 [1, 27, 28]. Vaccines composed of RBD are able to induce powerful neutralizing activity [27, 28]. Furthermore, antibodies on the transmembrane domain or the N-terminal domain are capable of neutralizing the virus primarily by interfering with protein rearrangement hence inhibiting the fusion process [27]. Figure 1 represents the structure of SARS-CoV-2 and the spike glycoprotein [17].

## SARS-CoV-2 variants

There are now five SARS-CoV-2 VOCs. This group includes the Omicron, Delta, Beta, Alpha, and Gamma which were originally identified in South Africa, India, South Africa, UK, and Brazil respectively [29, 30]. In the spike glycoprotein, these variants share a considerable number of mutations, with Omicron having an especially high number of mutations [29, 30]. These variants are further discussed in this article.

1. Omicron (B.1.1.529)

The World Health Organization (WHO) first identified this variant as a VOC in November 2021 [31, 32]. The spike glycoprotein of this variant is characterized by an extremely high number of mutations. Because the spike protein is the most common antigenic target for antibodies, the 32 mutations are expected to alter the effectiveness of vaccines that target the spike protein [31]. Even though the Delta variant only had 5 S protein mutations, it had worldwide consequences [31]. Mutations in the Omicron variant have been found in a variety of SARS-CoV-2 proteins, including NSP4, NSP14, NSP3, S protein, envelope protein, NSP5, NSP6, membrane protein, NSP12, and nucleocapsid protein [31]. Omicron has a large number of deletion mutations, totaling more than 30. Some of the alterations are similar to those found in Alpha, Gamma, Beta, and Delta VOCs. The 5 SARS-CoV-2 VOCs have the alterations N679K T478K, 69–70del, N501Y, K417N, N655Y, T95I, G142D/143–145del, and P681H [32]. Greater viral binding affinity, enhanced transmissibility, and higher antibody escape are all connected to distinct types of mutations [32].

Because of the abrupt increase in the number of mutations in the spike glycoprotein, it is possible that Omicron is caused by vaccination. As a result, the spike protein’s 32 amino acid modifications are likely to improve the variant’s ability to evade current vaccinations [31]. The virulence, infectivity, and ability of this mutant to evade vaccination protection are all unclear at this time [31].

2. Alpha (B.1.1.7)

It is one of the most frequent VOCs found in Canada. In September 2020, it was first reported in the United Kingdom. When compared to other VOCs, it has a 50% higher transmissibility [33]. AstraZeneca’s vaccine was shown to be 70% effective against alpha VOCs, according to the results of a research. In another trial, the Pfizer vaccine was found to be 93.7% effective against alpha VOCs [34].

3. Beta (B.1.351)

In the month of May 2020, this strain was discovered in South Africa. The VOC was associated with an increase in hospitalizations and deaths. As a result of beta VOC, current vaccines appear to be less efficient in suppressing COVID-19 infection, according to available evidence [33, 35]. According to the findings of a trial, a full dose of Pfizer vaccine was 75% effective against Beta variants. The Pfizer vaccine was 89.5% effective against Alpha variants. However, for severe disease from either the Beta or Alpha variants the effectiveness stood at 97.4% [35]. Novavax’s efficacy against Beta variants was 89% in the UK and 60% in South Africa, respectively. The trial results for the Johnson and Johnson vaccine revealed that it provided lower levels of defense in eradicating moderate to severe COVID-19 in South Africa than in the United States [35].

4. Gamma (P.1)

In the month of November 2020, this variant was discovered in Brazil. In comparison to the other prevalent variants in the country, it is 1.7–2.4 times more transmissible [35]. The spike protein’s mutations improve its ability to adhere to human cells. Some of the mutations it encompasses are similar to those found in Beta and Alpha VOCs. SARS-CoV-2 pre-exposure provides little to no protection against reinfection with Gamma variants. In comparison to the Beta VOC, the Gamma variant was less resistant to antibody reactions, according to the results of a preprint study. This was due to a previous illness or vaccine [35].

5. Delta (B.1.617.2)

In October of 2020, this variant was discovered in India [36]. It is characterized by a high level of transmissibility [35]. According to reports from UK studies, when compared to the Alpha variant, this variant is 60% more transmissible [35]. High rates of reinfection, higher viral load, and longer infection duration were all directly connected to the variant’s improved transmissibility. This is owing to the variant’s ability to evade natural immunity [32]. In comparison to other VOCs, this variant has had disastrous global impacts [32]. The emergence of new variants despite widespread vaccination raises questions about the efficacy of current SAR-CoV-2 vaccines [32].

The current vaccines on the market have shown to be less efficient against Delta VOCs than the Alpha variant, yet they are very beneficial for illness prevention after a full dose [35]. According to a study, the Pfizer vaccine was 88% and 93% effective against the Delta and Alpha VOCs, respectively, after a full dose. The vaccine’s efficacy against Delta and Alpha strains was 60% and 66% for AstraZeneca after two doses, respectively [35]. The effectiveness of the Pfizer vaccine was 94% after the first dosage and 96% after the second dose. However, after one dosage and two doses, the AstraZeneca vaccine was 71% and 92% effective against hospitalizations, respectively [35].

## SARS-CoV-2 mutations

The S protein identified in the initial Wuhanhu1 strain was incorporated into current COVID-19 vaccines made by Pfizer-BioNTech, Oxford-AstraZeneca, Sinovac, Sinopharm, Bharat Biotech, Gamaleya, Novavax, Johnson and Johnson, and Moderna [20, 37–39]. Concerns about the efficiency of neutralizing antibodies and cell-mediated immunity provided by existing vaccines have arisen as a result of the emergence of diverse strains. Omicron, Delta, Gamma, Alpha, and Beta are the names given to these VOCs [30, 31, 40–42].

SARS-CoV-2 is causing significant missense mutations in the spike glycoprotein at the moment. Virus transmissibility and virulence are predicted to rise as a result of the mutations, lowering the effectiveness of presently used vaccines [11, 12]. These mutations are clearly synthesized in this article and comprise of:

i. Substitutions in the spike protein: T478K, N501Y, A67V, T547K, T95I, Y145D, L212I, G339D, S373P, K417N, N969K, N440K, Q954H, S477N, E484A, Q498R, Y505H, N679K, N764K, N856K, S371L, del211, ins214EPE, del142-144, del69-70, S375F, L981F, H655Y, G446S, Q493R, G496S, D796Y, P681H, D614G
ii. The spike protein in the Omicron variant has been extensively mutated [31]. This strain has since been denoted as the Omicron variant (WHO nomenclature) and B.1.1.529 (PANGO lineage) [31, 43]. In comparison to other VOCs, the spike glycoprotein of the Omicron variant includes 26 amino acid alterations. There are two deletions, 23 replacements, and one insertion in this list. It is the first time in the SARS-CoV-2 lineages that an insertion mutation (ins214EPE) has been discovered [43]. Template switching could have resulted in the nucleotide sequence encoding the insertion mutation (ins214EPE). The human transcriptome of host cells already infected with the Omicron variant or other viral genomes capable of infecting the same host cells as SARS-CoV-2 could have been used to switch templates [43].
iii. L452R

This type of mutation can be identified in Delta VOC. The amino acid leucine (L) is changed to arginine (R) in this mutation [29]. This RBD mutation increases the affinity of the ACE2 receptor for binding and can reduce the interaction with vaccine-elicited antibodies [44]. It also enhances T cell resistance, which is responsible for identifying and eliminating virus-infected cells [29].

iv. D614G

The change from aspartic acid (D) to glycine (G) at position 614 is what gives this mutation its name. This mutation can be found in every VOC [45–47]. According to studies, this type of mutation is directly associated with enhanced transmission rate and infectivity, as well as the ability to cause sleeplessness [29, 45, 46, 48, 49]. The increased amount of spike glycoproteins per virion and the increased rate of S1/S2 cleavage could be due to this mutation [29].

xxii. K417

This mutation can be found in the spike glycoprotein’s RBD. The spike glycoprotein is involved in the virus’s interaction with the human ACE2 receptor protein [29, 50, 51]. K417N mutations are more common in the Beta strain, whereas K417T mutations are more common in the Gamma variants [29, 50, 51]. Reduced sensitivity to neutralizing antibodies and greater transmissibility are two characteristics of the K417T and K417N mutations [50, 51].

vi. E484K

The name comes from a substantial change in glutamic acid (E) to lysine (K) at position 484. A large frequency of polyclonal antibodies in SARS-CoV-2 infected people is frequently used to identify this type of mutation [29]. This mutation affects the Gamma and Beta VOCs [29, 52]. According to reports, the identical mutation can be found in several sub-lineages of the Alpha variant [29]. By boosting the virus’s ability to evade the immune system, the E484K mutation impairs the antibody recognition mechanism. As a result, there is a higher chance that this mutation will affect the efficacy of existing vaccines [52].

vii. N501Y

This mutation is seen in all VOCs and results in the amino acid asparagine (N) being changed to tyrosine (Y) at position 501. This mutation has a number of consequences, including increased virus replication in hamster upper respiratory tracts and human upper-airway cells and increased binding affinity to human ACE2 [29]. N501Y has the ability to cause larger concentrations of the virus in the nasal cavity and pharynx, resulting in a higher transmission rate [41].

viii. P681

The Alpha VOC has a P681H mutation, while the Delta VOC has a P681R mutation [29]. According to current data, the prevalence of P681H has continued to rise at an exponential rate. Increased viral fusogenicity and pathogenicity are associated with the mutation, which is located at the furin cleavage site [45]. Furthermore, as a result of immunization, the P681R mutation displays significant resistance to neutralizing antibodies [53].

## Classification of COVID-19 vaccine technology platforms

Inactivated viruses, mRNA, DNA vaccines, protein subunits, nonreplicating and replicating vector, VLP, and live attenuated vaccines are among the seven kinds of SARS-CoV-2 vaccines available [3, 8, 14, 54, 55]. Nucleic acid vaccines, which use fragments of the virus’ genetic material, are part of group A. In this kind of vaccine, individual bodily cells receive the viral genetic material directly. DNA and RNA segments are combined into a plasmid, which is then taken up by host cells and controls the entire process. The virus protein is then mass-produced within the host cells, triggering an immunological response [54, 56]. Vaccines with knocked-out viruses make up group B. Inactivated or weakened viruses are used in this class of vaccines. It is highly recommended that the viruses be entirely inactivated in this technique so that they do not cause illness [54, 56].

Vaccines with viral vectors make up group C. Adenoviruses are used in these vaccines to introduce transcribed DNA segments from SARS-CoV-2 into other viruses. The injected vectors are critical in training body cells to make coronavirus glycoproteins, which in turn triggers an immunological response [54]. The problem with this method is that if the genetic material coding for the antigen is lost, there is a risk of vaccine failure. This occurs during the vaccines’ manufacturing operations [56].

**Fig. 2:**
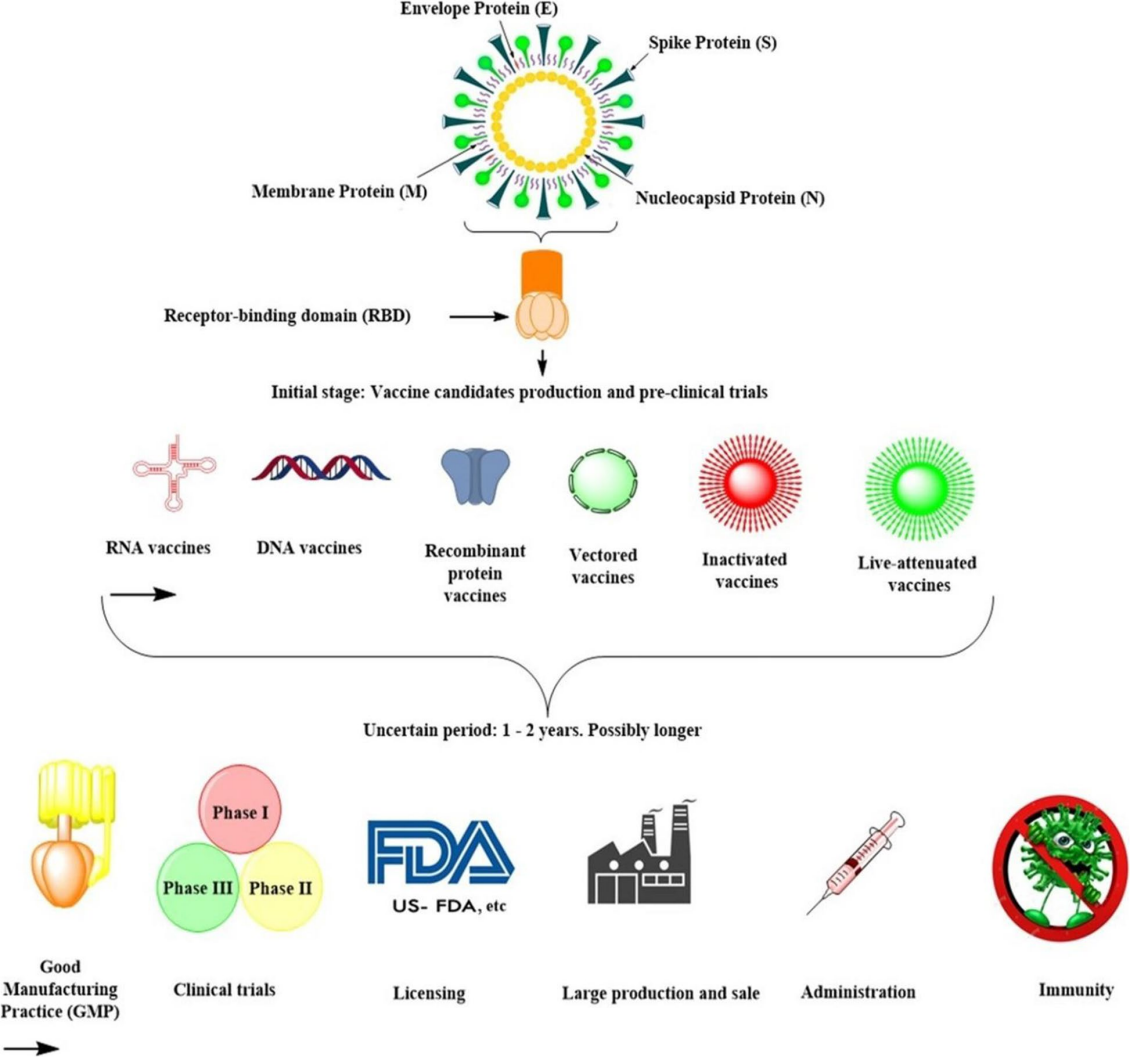
Schematic representation of the different types of vaccine technologies available [25]

The protein subunit vaccines make up group D. Virus proteins are used in the vaccines, either in whole or in parts. These proteins are encapsulated in nanoparticles for better distribution and absorption by human cells [54]. The protein subunit vaccines make up group E. The protein coronavirus protein subunits are produced and then combined to form VLPs with properties comparable to those of SARS-CoV-2. The DNA vaccines belong to group F. In this group of vaccines, the reverse transcription technique is used to create the vaccines from viral RNA. Attenuated and repurposed vaccines make up group G. These vaccines’ preparation and development are based on existing vaccine preparation techniques [57, 58]. The features of several types of vaccines that have been approved for usage are summarized here. Figure 2 depicts a schematic representation of the various vaccine technologies available [25].

## COVID-19 vaccine technology platforms

- mRNA Vaccines

These vaccines have good safety profiles, low manufacturing costs, strong immunogenicity, fast manufacture, inherent adjuvant qualities, and unique storage and administration systems [8, 59, 60]. The technology is quite advanced, and this is the first time it has been used on people. For many years, mRNA vaccine production technology has been studied for a variety of viruses, including Zika, rabies, and influenza [60, 61]. These vaccines, on the other hand, are the first COVID-19 vaccines to be licensed and used in humans. These vaccines offer a number of benefits, including the capacity to allow body cells to manufacture S proteins rather than injecting them [9, 62]. In comparison to the time required for traditional vaccines, this technique requires less time [9].

Manufactured modified-nucleoside, single-stranded mRNA is used to convey genetic instructions primarily to host cells. Encapsulated mRNA reaches human cells during this procedure [62]. Encapsulation protects mRNA from destruction by body cells and also stabilizes it, which is important because it is a very fragile molecule [8–10, 60]. These mRNA molecules persist in body cells for fewer than two days [1]. The mRNA can send instructions to the ribosomes in the cytoplasm of human body cells within this timeframe. Once the process is complete, the mRNA is digested by ribonucleases, which are a type of enzyme [8, 9, 60]. Because the risk of unanticipated long-term expression and genetic integration is eliminated, mRNA vaccines are safer. This is due to the fact that mRNA does not cross through the nucleus [14, 62]. Furthermore, the technique has the benefit of mRNA cell-free generation, which reduces bacterial contamination. The process is characterized by cheap manufacturing costs and enables rapid scale-up [60].

The initiation of adaptive immune responses is triggered by the production of S protein by body cells. Cell-mediated immune responses and humoral responses are two types of adaptive immune responses [8, 9]. The neutralizing antibodies produced during the humoral responses have the ability to prevent the spike protein from binding to the ACE2 on the host cells. The killer T cells then identify contaminated cells and kill them [1, 60]. This includes Pfizer–BioNTech Vaccine (PBV), Moderna Vaccine (MV), and CureVac’s CVnCoV Vaccine (CVV). This article summarizes the distinctions between these vaccines.

i. Pfizer–BioNTech vaccine

Pfizer and BioNTech, based in New York and Germany, respectively, manufacture this vaccine [63]. This vaccine is packed as a lipid nanoparticle and works against the SARS-CoV-2 virus’s spike glycoprotein [1, 63]. This method employs genetically engineered RNA to produce a protein capable of inducing rapid immunological responses [1]. The vaccine primarily works by helping the body to produce antibodies that neutralize the pathogen. For the virus to enter alveolar cells via the ACE2 receptor, it is completely reliant on the spike protein [7, 63]. The vaccine is given in two doses separated by 3 weeks [8, 64]. According to manufacturer reports, the vaccine is 95% effective [1, 7, 8, 10, 65]. The vaccine’s effectiveness for severe illness, on the other hand, was 87.5% [1].

The vaccine does not have any major negative effects; therefore, it can be given to everyone. Soreness at the injection site, weariness, muscle pain, and a moderate fever are all common symptoms. All of these symptoms are frequently temporary [1]. This vaccine can only be stored at − 70 °C, this limits the usage of the vaccine in remote settings and certain countries [1, 62].

ii. Moderna vaccine

This vaccine was developed by Moderna. The vaccine can be kept at a temperature of − 20 °C, allowing for its shipment and utilization in remote and rural settings [1]. Data from the manufacturers shows that the efficacy of the vaccine is 94.5% [1, 7, 10, 62] It is suitable for individuals who are 18 years and above. The vaccine is administered in 2 doses 4 weeks apart. This vaccine is safer as no safety concerns have been raised [1, 8].

**Fig. 3:**
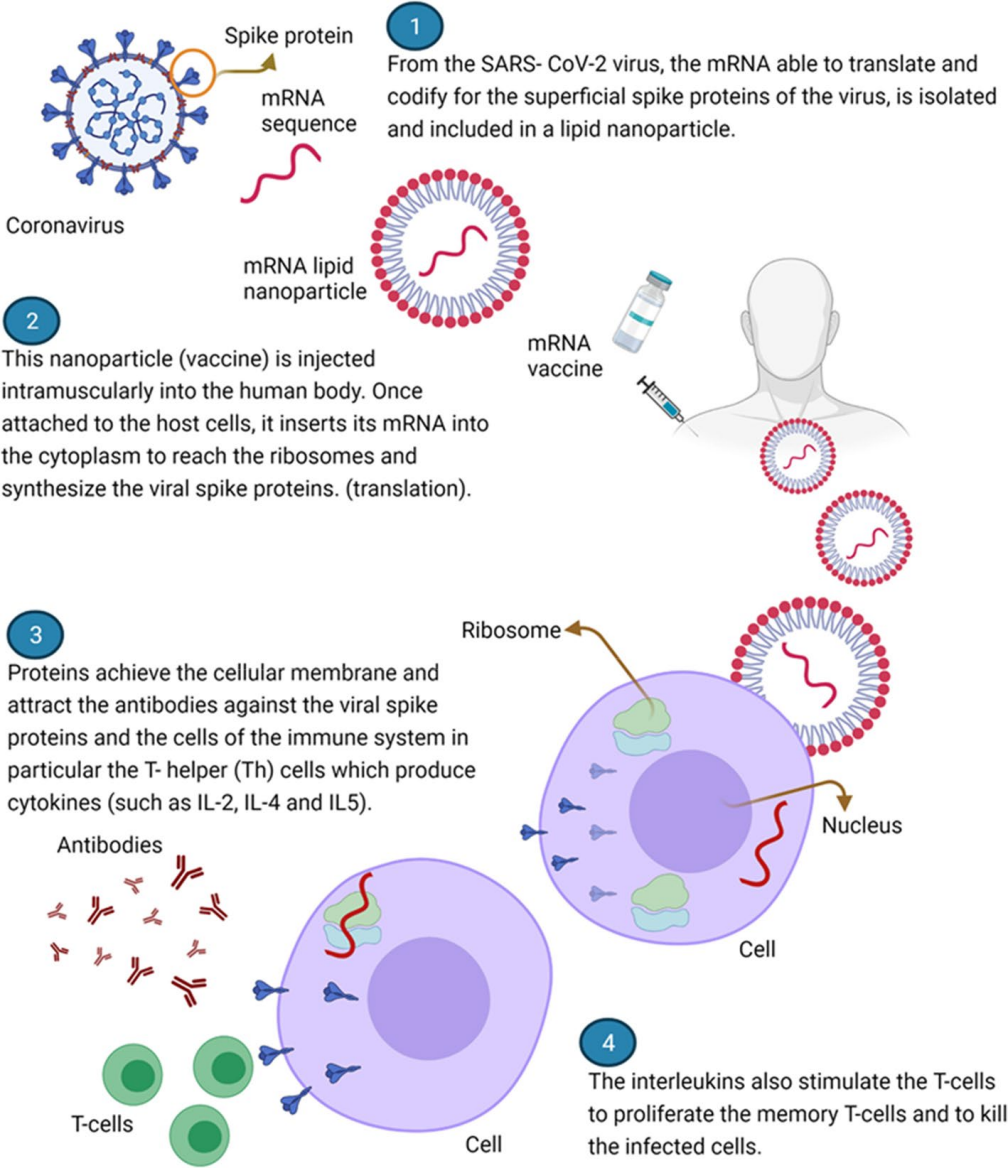
Represents a summary of Moderna and Pfizer-BioNTech vaccines’ mechanism of action [1]

Moderna vaccine induces vigorous binding and neutralizing antibody response. At the same time, the cell-mediated immune responses are activated resulting in the elimination of the virus by the cytotoxic T cells [7]. Results from a study conducted in Qatar indicated that the Moderna vaccine was efficacious against Beta and Alpha VOCs [66]. Figure 3 represents Moderna and Pfizer-BioNTech vaccines’ mechanism of action [1].

iii. CVnCoV vaccine

This vaccine was manufactured by CureVac biotech firm in association with Bayer Pharmaceutical Company. The vaccine is competing with the Moderna and Pfizer–BioNTech vaccines [1]. In this technology, a natural, non-chemically modified synthetic mRNA is utilized. This mRNA encodes the full-length S glycoprotein [1, 67]. The vaccine is administered intramuscularly. A complete dose is composed of two dose regimens, given 4 weeks apart [67]. This vaccine can be stored at 5 °C and can be stable for a period of 3 months if stored at a temperature of between 2 and 8 °C hence making it easy for it to be distributed and utilized in poorer countries [1].

b. Human adenovirus replicating and nonreplicating vector-based vaccines

These vaccines use either attenuated replication-competent viral backbones or replication-deficient viral vectors [7, 68]. The vectors utilized can either be replicating or non-replicating [7]. Currently, adenoviruses are heavily employed in the transportation and delivery of a selected plasmid. The plasmid contains a double-stranded DNA portion of the SARS-CoV-2 RNA which encodes the Spike glycoprotein [7, 8]. After vaccination, the immune system is usually active and is able to attack SARS-CoV-2 virus in case its encountered [7].

The adenovirus vectors comprise of the human Ad26 and Ad5 adenoviruses and a chimpanzee adenovirus ChAdOx1 [7]. After injection, the vectors are able to go into the body’s cells though they do not replicate intracellularly. The genetic material escapes from the vectors and travels straight to the nucleus. It is in the nucleus that the DNA is stored. The genetic material is thereafter transcribed into mRNA which escapes from the nucleus to be read and “translated” into spike proteins. The proteins are then assembled on the infected cell surfaces. Once the Spike glycoproteins are recognized by the immune system, the immune system generates specific neutralizing antibodies followed by T cells activation. The activated T cells destroy the S protein [7, 62]. Examples of vaccines that fall under this group include Oxford–AstraZeneca Vaccine, Sputnik-V Vaccine, Johnson and Johnson Vaccine, and AD5-nCoV Vaccine [68].

i. Oxford–AstraZeneca vaccine

This vaccine was manufactured by Oxford University in association with AstraZeneca [7, 10, 62]. This vaccine is often referred to as ChAdOx1 nCoV-19. It is a nonreplicating adenovirus vaccine vector (ChAdOx1) derived from a chimpanzee [7]. It employs a modified chimpanzee DNA adenovirus, that does not produce an immune response to the adenovirus itself, but to the viral protein which is encoded in the host DNA [1, 10].

The overall efficacy of the vaccine stands at 75% [10, 62]. Vaccine administration is composed of two doses, 28 days apart. The gap between the initial dose and the second dose was increased to 8–12 weeks for better efficacy [8]. Early studies indicated that this vaccine is generally safe with the exception of mild effects including fatigue, headache, pain at the point of injection, myalgias, redness, and arthralgias [1]. However, fears continue to circulate in relation to the potential side effects associated with this vaccine utilization including thromboembolic events [1]. The vaccine can be kept at 2–8 °C for a period of 6 months. This provision makes it easy for the vaccine to be stored, transported, and distributed globally [1].

**Fig. 4:**
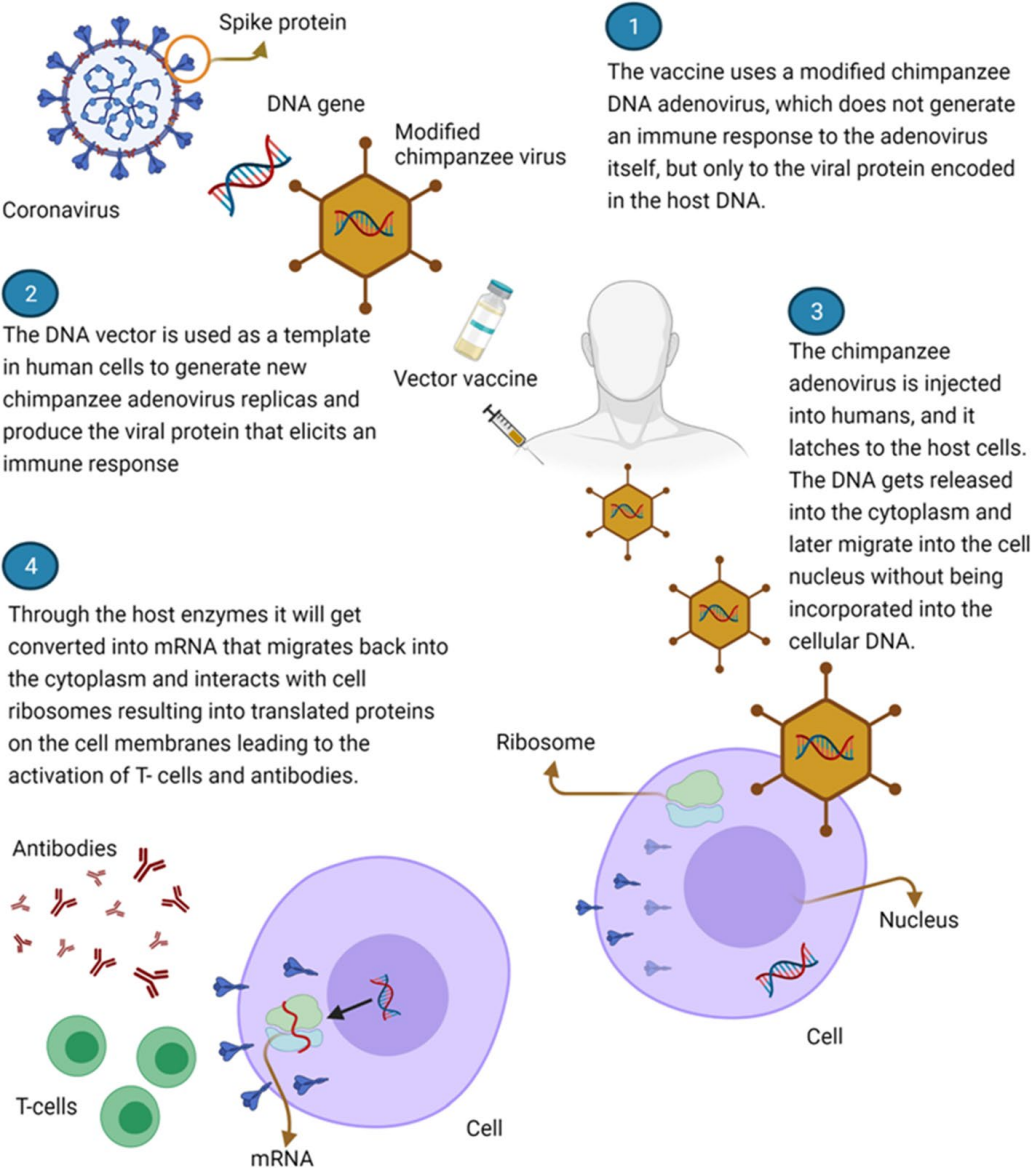
Represents a summary of the mechanism of action of AstraZeneca vaccines [1]

The name of the vaccine was changed from AstraZeneca vaccine to Vaxzervria with the approval of the European Medicine Agency in March 2021 [1]. Data from the UK study showed that the AstraZeneca vaccine is 74.5% and 67.0% effective against the Alpha and Delta variants respectively [69]. The same vaccine confers 77.9% and 10.4% protection against Gamma and Beta VOCs respectively [70, 71]. Figure 4 represents a summary of the mechanism of action of AstraZeneca vaccines [1].

ii. Sputnik-V vaccine

It is a Russian vector-based vaccine, produced by Gamaleya Institute [10, 62]. The vaccine development technology is based on Ad 26 and Ad 5 which are capable of stimulating a stronger and long-lasting immune response as compared to vaccines employing the same vector in two different doses [1, 62]. The vaccine is relatively cheap as compared to the other vaccines in the market. The immune system does not recognize both Ad5 and Ad26 as foreign and therefore they are not destroyed [72, 73].

In this vaccine platform, Ad26 and Ad5 are used as vectors for the expression of the coronavirus spike glycoprotein [1, 8, 10]. Two varying serotypes are used in order to overcome challenges arising as a result of pre-existing adenovirus immunity within the population [1].

The vaccine has an efficacy of 91.6% in protection. This is after a complete dose which is comprised of 2 doses given 3 weeks apart intramuscularly [8, 62, 72]. Ad26 vector is used in the first dose and Ad5 vector is utilized in the second dose [1]. These doses are given 21 days apart. The vaccine can be kept at − 20 °C [1, 8].

iii. Johnson and Johnson vaccine

This vaccine was developed by Janssen Pharmaceutical. The Company belongs to Johnson and Johnson Multinational Corporation [62]. In this technology, Ad26 adenoviral vector is used [62]. The vaccine was approved for use in individuals 18 years and above in February 2021 [1].

The vaccine technology uses adenovirus 26 CoV2 in delivering a gene carrying the blueprint for the S glycoprotein which is found on the coronavirus surface [74]. The vaccine is only administered once and is capable of producing a stronger neutralizing antibody response in at least 90% of vaccinated individuals after 4 weeks and after 2 months in all the recipients [1, 8, 10, 62]. This vaccine can as well be kept at 2–8 °C for up to 3 months. Furthermore, the vaccine can as well be kept at − 20 °C for a period of 2 years [1, 8]. This vaccine is 66% effective in preventing disease after a single dose and is capable of suppressing 85% of severe COVID-19 illnesses within 28 days post vaccination [1, 7, 8, 62]. The vaccine is also highly effective against the B.I.351 lineage observed in South Africa [1].

iv. AD5-nCoV vaccine

This vaccine was manufactured by the Chinese CanSino Biologics Company in association with the Academy of Military Medical Sciences [10, 62]. It uses the Ad5 adenovirus vector [8, 62]. Only one dose is administered and its efficacy is 65.28% [62]. Data collected from the phase 1 clinical trial suggested that this vaccine is safer, is tolerable, and is able to induce both humoral and cellular responses [75].

iii. Inactivated coronavirus vaccines

These vaccines are fully recognized by the immune cells, resulting in a powerful immune response. The vaccines against hepatitis A, influenza, and rabies are examples of inactivated virus-based vaccines created to stimulate a proper response against such pathogens [10]. Currently, several COVID-19 vaccines based on inactivated viruses are being developed. These vaccines are the CoronaVac, Sinopharm, Covaxin, and Sinopharm–Wuhan vaccine [10].

i. Sinopharm vaccine

This vaccine was manufactured by the Sinopharm Group. In this technology, Vero cell–cultivated and inactivated forms of the virus are used. The vaccine has a vaccine vial monitor for the detection of vaccine safety [8]. The Sinopharm vaccine is given in a 2-dose regimen, given 21 days apart by intramuscular injection [8]. The efficacy of the vaccine is 79.34% in China, though it is 100% effective in suppressing moderate to severe COVID-19 cases [8].

ii. Sinopharm–Wuhan vaccine

This vaccine was manufactured by the Chinese Wuhan Institute of Biological Products [8]. This vaccine technology makes use of the WIV-04 strain that was first isolated and then cultivated in a Vero cell line for propagation [76]. Later, the infected cell supernatant was inactivated using β-propiolactone, then mixed with an aluminum-based adjuvant [76].

iii. CoronaVac vaccine

The vaccine was developed by SinoVac Biotech in association with the Brazilian research center. It is a patient-derived SARS-CoV-2 virus strain, grown in the Vero cell line and eventually inactivated with beta-propiolactone treatment [10]. In this technology, an inactive virus is used as an antigen. An inactivated form of the virus is used in the generation of an immune response [1, 10].

iv. Covaxin vaccine

This is a type of vaccine that was prepared by the Indian Bharat Biotechnology Company. It is a Vero cell–based whole-virion SARS-CoV-2 vaccine [77]. This vaccine is used in India only in emergency situations. Phase 3 trial results showed that the vaccine was 77.8% efficacious against symptomatic cases and it offered 65.2% protection against Delta VOCs [78].

iv. Recombinant protein subunit vaccines

These vaccines use nanoparticles that contain complete or fragments of viral proteins [79, 80]. Because the vaccine does not employ genetic material, it cannot cause disease. Pre-clinical trials are underway for five candidate vaccines in this category. Different protein components are used in each vaccine [81]. The protein subunits can primarily induce specific neutralizing-antibody responses and T cell activation [79]. Novavax vaccine, EpiVacCorona vaccine, and ZF 2001 (RBD Dimer) vaccine fall under this.

i. Novavax vaccine

The vaccine was prepared by Novavax in association with GSK and Sanofi. This process was achieved through attachment of viral proteins onto the nanoparticle carrier hence facilitating efficient delivery and uptake by human body cells [82]. The vaccine technology uses harmless protein fragments which mimic the SARS-CoV-2 virus spike glycoprotein to generate an immune response [1]. This vaccine contains an adjuvant whose role is to strengthen the immune response [1]. It is administered intramuscularly and it consists of 2 doses which are given 3 weeks apart. This vaccine is capable of producing a strong antibody response and activating the T cells [83]. Novavax vaccine is very stable at refrigerator temperatures. Clinical trials conducted in the UK indicated that the efficacy of Novavax was 89.7% [1].

ii. EpiVacCorona vaccine

It was manufactured by the Vector Institute [10]. Its technology is based on pieces of synthetic viral peptides reflecting SARS-CoV-2 antigens. 3 chemically synthesized peptides of the S glycoprotein, expressed as a chimeric protein are utilized [10]. The vaccine is administered intramuscularly in 2 doses 3 weeks apart. People aged 18 years and above are eligible for this vaccine [83].

iii. ZF 2001 vaccine

This vaccine was developed by the Chinese Anhui Zhifei Longcom in collaboration with the Academy of Military Medical Sciences [10]. It was developed with the ultimate goal of targeting the RBD ( dimeric form) of the SARS-CoV-2 spike protein as antigen [84]. Results of phases I and II showed that the vaccine protein subunit was well tolerated and highly immunogenic [10]. In this vaccine technology, a section of the spike protein referred to as the RBD combined with an adjuvant is used [10]. The administration regimen consists of 3 doses given after every 4 weeks. It is injected intramuscularly [10].

e. VLP vaccines

The technology utilizes VLPs, which are self-assembled viral structural proteins capable of mimicking the structure of natural viruses though they lack the viral genome [8, 18]. These vaccines present epitope in a manner that is similar to the natural virus resulting in enhanced immunization responses [18]. This technology is advantageous as the production of these vaccines does not depend upon inactivation steps or live viruses [18]. The highly repetitive antigenic surface of VLP vaccines produces a stronger antibody response by effectively cross-linking B cell surface receptors [18]. Currently, these vaccines are being used in the protection against hepatitis B virus and human papillomavirus [18].

These vaccines depend upon adjuvants and repeated administration for them to elicit a stronger immune response. VLP vaccines technology utilizes non-infectious VLPs similar to SARS-CoV-2 particles both in structure and morphology, though they lack infective genetic materials [85–87].

f. Repurposed and live attenuated vaccines

The Bacillus Calmette–Guerin vaccine is an example of a live attenuated vaccine developed primarily for the prevention of tuberculosis [3, 88]. Several vaccines of this kind are under preclinical trials in Turkey and India [17]. Another example of a vaccine manufactured using this technology is COVI-VAC. The vaccine was prepared by the Serum Institute of India in partnership with Codagenix [89]. This technology is robust and can be engineered to produce vaccines that can recognize the whole virus and be administered via the intranasal route [17].

## Conclusion

The COVID-19 pandemic is one of the world’s most lethal and contagious diseases. Vaccines are being produced all throughout the world with the ultimate goal of combating the disease. However, as time goes on, more SARS-CoV-2 mutations appear. Vaccines based on non-replicating viral vectors and RNA now have very high efficacies, giving the world hope that recovery is on the horizon. As patients around the world receive these vaccines, no information on possible long-term negative effects from any of the anti-SARS-CoV-2 vaccines currently in use is available. Despite the fact that the genomes of SARS-CoV-2 are not as variable as those of other viruses, the RBD located in the spike glycoprotein is the most mutable area, as seen in the Omicron variant. Increased infectivity and lower antibody binding are linked to these alterations. The effectiveness of the currently available SARS-CoV-2 vaccines is anticipated to be hampered as a result of the increasing mutations on the spike glycoprotein. As a result, vaccine candidates capable of eliciting a large antibody repertoire as well as a robust cellular immune response are clearly attractive, as they may provide more long-lasting and broad protection. Finally, sequencing each SARS-CoV-2 genome is critical because it will give a variant warning system that will allow for early detection of variants of concern based on their mutational profile.

## Data Availability

Not applicable.

## Abbreviations

SARS-CoV-2: Severe acute respiratory syndrome coronavirus 2
ACE2: Angiotensin-converting enzyme 2
mRNA: Messenger RNA
RBD: Receptor-binding domain
RdRp: RNA-dependent RNA polymerase
VOCs: Variants of concern
VLP: Virus-like particles
